# Ultrafine Kaolinite Removal in Recycled Water from the Overflow of Thickener Using Electroflotation: A Novel Application of Saline Water Splitting in Mineral Processing

**DOI:** 10.3390/molecules28093954

**Published:** 2023-05-08

**Authors:** Felipe M. Galleguillos Madrid, María P. Arancibia-Bravo, Felipe D. Sepúlveda, Freddy A. Lucay, Alvaro Soliz, Luis Cáceres

**Affiliations:** 1 Centro de Desarrollo Energético Antofagasta, Universidad de Antofagasta, Antofagasta 1240000, Chile; 2 Departamento de Química, Universidad Católica del Norte, Antofagasta 1240000, Chile; 3 Departamento de Ingeniería en Minas, Universidad de Antofagasta, Antofagasta 1240000, Chile; 4 Escuela de Ingeniería Química, Pontificia Universidad Católica de Valparaíso, Valparaíso 2374631, Chile; 5 Departamento de Ingeniería en Metalurgia, Universidad de Atacama, Copiapó 1531772, Chile; 6 Departamento de Ingeniería Química y Procesos de Minerales, Universidad de Antofagasta, Antofagasta 1240000, Chile

**Keywords:** kaolinite clays, electroflotation process, saline electrolyte, titanium electrodes, saline water splitting

## Abstract

The presence of ultrafine clay particles that are difficult to remove by conventional filtration creates many operational problems in mining processing systems. In this work, the removal of clay suspensions has been investigated using an electroflotation (EF) process with titanium electrodes. The results show that EF is a viable and novel alternative for removing ultrafine particles of kaolinite-type clay present in sedimentation tank overflows with low salt concentrations (<0.1 mol/L) in copper mining facilities based on the saline water splitting concept. Maximum suspended solid removal values of 91.4 and 83.2% in NaCl and KCl solutions, respectively, were obtained under the experimental conditions of the constant applied potential of 20 V/SHE, salinity concentration of 0.1 mol/L, and electroflotation time of 10 and 20 min in NaCl and KCl solutions, respectively. Furthermore, the visual evidence of particle aggregation by flocculation during the experiments indicates a synergy between EF and electrocoagulation (EC) that enhances the removal of ultrafine particles of kaolinite.

## 1. Introduction

Clays are minerals of the phyllosilicate type, formed mainly by alterations and weathering at low temperatures and pressure, which are intimately associated with the gangue or matrix rock so that their presence is inevitable in mining processing [1]. A common type of clay is kaolinite, chemically represented as an aluminum silicate with a layered crystalline structure (Al_2_Si_2_O_5_(OH)_4_) [2]. The presence of kaolinite in the processing of copper sulfide minerals is relevant in the mining industry, as it affects the entire value chain of the process [3]. Among the adverse effects caused by kaolinite, three stand out: (i) increase in fines in the size reduction stages, since this type of mineral can achieve sizes < 20 µm [1]; (ii) reduction in the grade of the copper concentrate, by shielding the bubbles, modifying the rheology of the foam, and impairing the selectivity of the flotation process [4], and (iii) reduction in the recirculated water quality from the overflow of the sedimentation process at dewatering plants due to the accumulation of suspended particles when operating at alkaline pH values (pH > 8). This condition leads to pyrite depression [5] and also to the modification of the kaolinite edge and face charges promoting particle dispersion and stability of the suspensions [6].

From a global perspective, water is the medium through which minerals are transported and processed in copper mining facilities. Thus, under conditions of scarcity of water, its rational use and recycling are mandatory. In this context, water quality significantly impacts operational efficiency, as described above [3], as well as environmental and economic aspects [7]. From an environmental point of view, recycling water within a mining processing plant can significantly reduce water consumption by using the same water to perform several mining operations and, in turn, reduce pumping operational costs [8,9,10].

Currently, the way to mitigate the negative impacts of clays in recirculated water is through (i) the use of flocculants [11], (ii) mechanical removal from hydrocyclones [12], (iii) dissolved air flotation (DAF) [13], and (iv) the use of dispersants [14]. Particle aggregation and the precipitation of some ions, such as magnesium carbonates and sulfates [15], are produced due to chemical additives in these techniques.

The electroflotation process (EF) is a technique to remove suspended particles and metal ions from wastewater [16,17,18,19,20,21,22] under a fixed composition of water which has been applied in many industries but until now has not been considered in the mining industry for the removal of ultrafine clays (colloids) present in saline electrolytes [23,24,25,26]. However, the removal of kaolinite or clays from different effluents without Cl^−^ ions present in the electrolytes has been studied using EF and EC techniques [27,28,29]. In Chile, using saline electrolytes or natural seawater in mineral processing is a reality, for which is necessary to provide information about the performance of the process under this new operational condition [30]. EF has also successfully removed organic particles in wastewater and used oils in the coal recovery processes, among other applications [31,32,33]. The removal of fine particles such as clays is sustained by floating them to the water’s surface with tiny bubbles of H_2_ and O_2_ generated from water electrolysis; a bubble diameter between 15 and 105 μm is an ideal range for a high probability of collision with the mineral [23,32]. Among its advantages are (i) the non-use of chemicals [34], (ii) the flow of bubbles can be easily controlled by adjusting the electrode potential, (iii) the low residence time, and (iv) the easy operational control [35,36]. Regarding the EF of minerals, there are only available reports on the removal of pyrite minerals [37] and chalcopyrite [35,38,39], all using electrolytes without the presence of salts. Other investigations have considered salts such as NaCl in hematite minerals [40], but without comparing it with other types of monovalent salts such as KCl. The importance of studying the EF process in saline systems lies in the increased use of seawater to supply the water deficit faced by copper-producing countries [30]. Aspects such as the effect of salinity on the solid separation efficiency and electrode integrity are the main problems to be investigated.

The efficiency for particle removal achieved by electroflotation relies on a sequence of steps associated with particle stability which in turn is governed by the hydrophobicity of the suspended particles [41]. In this sense, electroflotation may be implemented in several ways; if inert electrodes are used, only H_2_ and O_2_ bubbles are generated unless some impurities are present in water, and other reactions may take place; e.g., in the presence of chloride (Cl^−^), chlorine gas bubbles (Cl_2_) are released at the anode electrode. Otherwise, if the active electrode is used, aluminum (Al) or iron metal (Fe) ions are liberated into the solution to produce hydroxide ions that act as coagulants and enhance the removal efficiency of the EF process [5]. Ti, unlike Al and Fe, has a more positive oxidation potential in comparison to that of O_2_ evolution reaction (OER). Additionally, to achieve its electrochemical dissolution, a significant overpotential is required. As shown in Pourbaix diagrams for Ti in saline media at a neutral pH [42,43], three distinctive regions are noted: (a) an immune region at potentials more negative than −2.0 V/SHE (standard hydrogen electrode), (b) a passive region with predominant TiO_2_ which is formed as a very thin film of great stability, and (c) an anodic region with the formation of mainly TiO_2_^2+^ and TiO_3_·H_2_O at potentials more positive than 1.5 V/SHE. For this reason, depending on the applied potential, the EF process can be carried out either in the absence or in the presence of metal ions. This is an interesting feature to rapidly determine whether or not the assistance of a coagulant is needed to enhance particle removal.

The role of the electrochemical reactions that take place in the electroflotation, namely, hydrogen evolution reaction (HER), oxygen evolution reaction (OER), and Ti electrode dissolution are discussed in terms of the mechanisms and operational factors influencing the ultrafine clay particles removal process. This work aims to investigate the effect of monovalent cations such as Na^+^ and K^+^ in the modification of the superficial conditions of the kaolinite during the removal process and the effect of Cl^−^ ions present in the electrolyte. The electrodes were made in Ti mesh as cathodic and anodic electrodes, respectively, for the improvement in the EF process in mineral processing [44].

## 2. Materials and Methods

### 2.1. Electroflotation System and Materials

**Figure 1:**
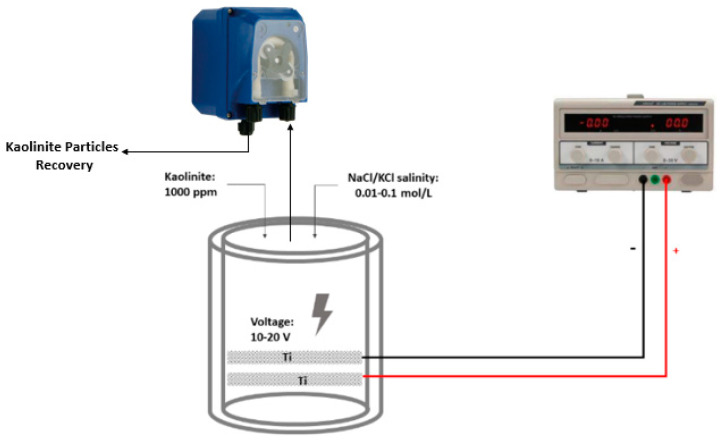
Schematic diagram of electroflotation cell.

The EF cell used in this work was a 250 mL volume cylindrical column fitted with two 5.25 cm-diameter titanium mesh cylindrical electrodes from Balance World Inc. (Putian, China). The effective area for each electrode was 13 cm^2^, as shown in Figure 1. Synthetic overflow water was prepared with 1000 ppm kaolinite in 0.1 mol/L NaCl solution. The electroflotation system was operated at room temperature using a power supply operating at a constant potential of 10 and 20 V/SHE with an operating time of 10 and 20 min. Three operating variables were considered for kaolinite removal: (i) *X*_1_, the applied potential in *V*, (ii) *X*_2_, the cell operation time in min, and (iii) *X*_3_, the salinity concentration in mol/L.

### 2.2. Minerals Characterization

The high-purity kaolinite sample, purchased from Science Words (Los Angeles, CA, USA), was characterized using a scanning electron microscope (SEM) (Hitachi SU 500 model, Ibaraki, Japan) and X-ray diffraction analysis (XRD) (Bruker advance d8 model, Billerica, MA, USA). Saline solutions of 0.1 mol/L NaCl and KCl were prepared using NaCl and KCl Merk analytical-grade reagents. The working pH was adjusted using NaOH and HCl, Merck analytical grade. Synthetic solutions were prepared with deionized water with a resistivity of 18.2 mΩ/cm.

### 2.3. Electroflotation Procedure

The simulated thickener overflow solution was prepared using a 0.1 mol/L NaCl solution dosed with 80% −5 µm particle size kaolinite clay (−635 mesh Ty) to achieve a concentration of 1000 ppm, as suggested by [15]. Pulp conditioning was 5 min at 900 rpm, establishing a fixed pH (pH = 8). For EF, using a magnetic stirrer, the homogenization time of the saline solutions was 15 min at 700 rpm. Then, the suspension was transferred to the reactor and energized according to the test number. For each solution, eight different runs were made to cover all combinations of operational factors listed in Table A1. At the end of a run, the froth layer was fully removed using a small pump and deposited in a beaker, then filtered and dried at 105 °C for 12 h with subsequent mass measurement in an analytical scale. Additionally, the remaining solution’s total suspended solids (*TSS*) (solution below the froth layer) were measured with a HACH model 2100. The *TSS* of the influent solution was calibrated to 1000 ppm for all runs. Considering that the froth layer at the end of a run may be composed of both clay particles and titanium hydroxides, the solid removal efficiency was determined in two different ways:

**(a)**  **The feed and tail effluent and effluent TSS measurements:**
  (1) $$R_{TSS}(TSS\;removal\;\mathrm{efficiency},\%)=\frac{X_{o}-X_{e}}{X_{o}}\cdot 100$$
  where *X_e_* and *X_o_* represent the feed and tail effluent *TSS* measurements, respectively.
**(b)**  **Based on mass froth collected:**
  (2) $$R_{MF}(mass\;removal\;\mathrm{efficiency},\%)=\frac{M_{1}}{m_{o}}\cdot 100$$
  where $M_{1}$ is the total solid mass measured in the froth, which includes Ti flocs and clays, and $m_{o}$ is the total mass of clay initially contained in the reactor.

The rationale of these two definitions is that in the complete absence of Ti flocs, both *R_TSS_* and *R_MF_* values should be the same, whereas when Ti flocs are present and are efficiently removed by bubbles, *R_TSS_* values must be lower than those of *R_MF_*. Increasing differences between *R_TSS_* and *R_MF_* should be observed for increasing Ti flocs. Additionally, different behaviors should be observed if the sedimentation of a fraction of particles or flocs takes place. The levels considered in this study are shown in Table A1 in Appendix A.

### 2.4. Zeta Potential Measurements

A Zeta-Meter 4.0 model from Zeta-Meter Inc. (Harrisonburg, Virginia) was used to measure the electrophoretic mobility and zeta potential of kaolinite. The solutions of 0.01 mol/L of NaCl and KCl in deionized water were used at pH 3 to 12. The mass of kaolinite was 100 mg with a size of 80% −20 µm (−635 mesh) in 50 mL of solution. According to the corresponding test, the conditioning time was 15 min at 700 rpm. Ten measurements for each trial were considered for the average calculation of the zeta potential.

## 3. Results and Discussions 

### 3.1. Mineralogical Characterization of Kaolinite

**Figure 2:**
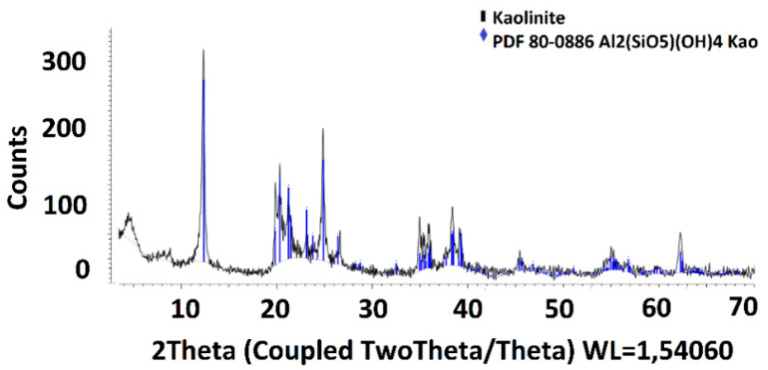
XRD analyses of kaolinite.

**Figure 3:**
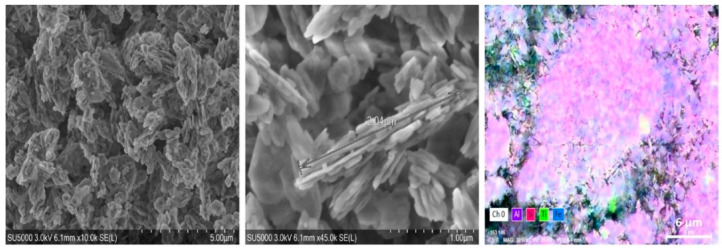
SEM and EDX analyses of kaolinite, where: the white color: oxygen; the purple color: aluminum; the magenta color: silicium; the green color: titanium; and the blue color: iron.

The kaolinite was formed from 44.53% Al, 48.57% Si, 4.44% Ti, and 2.47% Fe and had high purity (>98%). The XRD and SEM analyses are presented in Figure 2 and Figure 3. 

### 3.2. Zeta Potential of Kaolinite

Kaolinite surfaces immersed in freshwater are highly hydrophilic and coated with a layer of immobilized interfacial water molecules. Its improved flotation in saline water compared to fresh water has been attributed to a decrease in the absolute value of the zeta potential of particles. A significant zeta potential change toward more positive values with increasing NaCl and KCl solution concentrations between 0.01 and 0.1 mol/L has been demonstrated [45].

**Figure 4:**
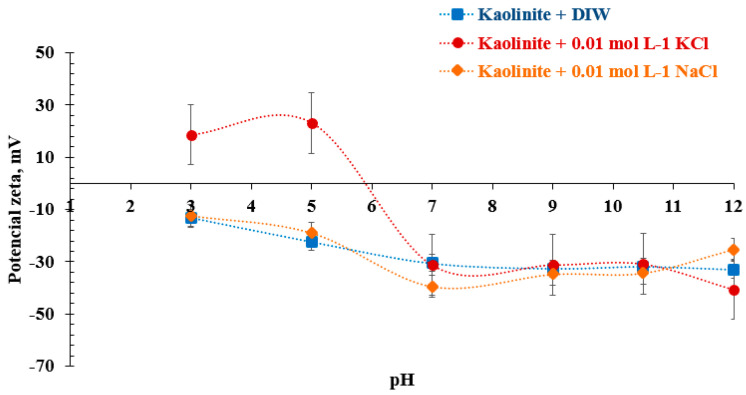
Zeta potential of pure kaolinite as a function of pH in saline solutions of NaCl and KCl.

The differences generated in the potential of kaolinite when studied with 0.01 mol/L solutions of NaCl and KCl are shown in Figure 4. Only this concentration was considered because, at a higher molarity, the ionic interactions would affect the reading of the equipment [46]. In the case of *DIW* (deionized water) and NaCl, the values are similar throughout the pH range and are consistent with the values previously reported by Ma et al. [45]. In the case of the KCl effect, its potential is affected by pH, as seen in Figure 4 (red line). At an acidic pH, kaolinite shows a potential change in the presence of K^+^ ions with positive values at a low pH, revealing that the face and edge of the mineral are negatively and positively charged, respectively [47]. This is due to the nature of this type of ion, considered a breaker of water structures (breaker ion) [45]. On the other hand, the Na^+^ ion promotes the hydrated layer on the kaolinite due to its tendency to generate aggregates with each other, probably negative, which promotes its tendency to stabilize in suspension in the medium [48].

The zeta potential measurements performed in 0.01 mol/L of NaCl and KCl solutions that agree with previously reported values [45] are shown in Figure 4, and the values determined by the zeta meter are presented in Table A2 in Appendix A. Considering that the recovered kaolinite is an innocuous waste of the process, its zeta potential determination is necessary to know its hydrodynamic behavior [49,50]. Three main observations from this figure are (i) the zeta potential of kaolinite decrease with increasing pH in both solutions, (ii) in the presence of KCl, the isoelectric point (*IEP*) takes place at pH close to 6, and (iii) no significant effect on zeta potential is observed at alkaline pH values (pH > 7). At the working pH (pH = 8) in both working solutions, the typical values (−30 mV) are consistent with earlier reports, values that were reproduced by Zheng et al., among others [48,51,52].

### 3.3. Effect of Low Saline Concentration Electrolyte over EF Process

**Figure 5:**
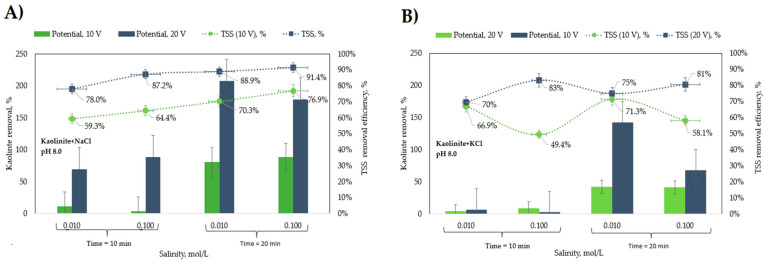
Effect of the (**A**) NaCl and (**B**) KCl concentrations on the kaolinite removal.

**Figure A1:**
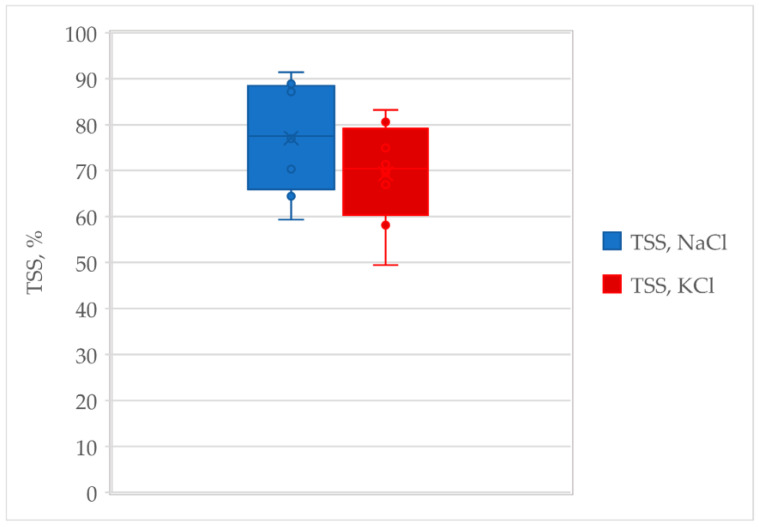
Bar error plots for NaCl and KCl solutions.

**Figure A2:**
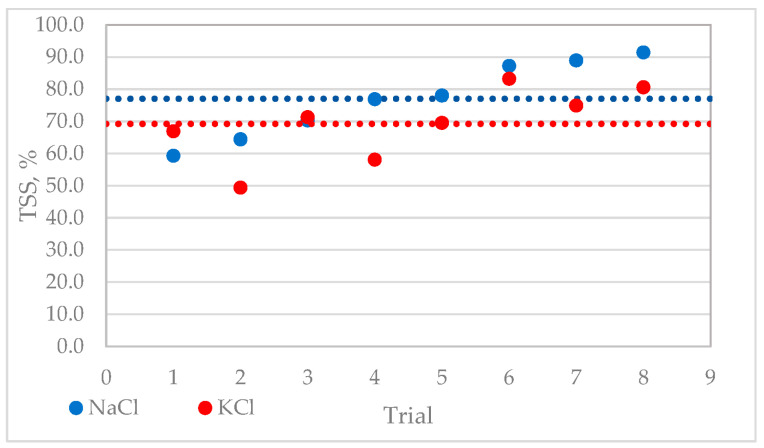
%TSS tendency for NaCl and KCl solutions.

A swarm of small bubbles emerging from the electrodes and floc formation was evident during all the experiments. This allowed the particle–bubble aggregates to rise into the froth layer that steadily grew with time. As the applied potential largely exceeds the standard potential of water and titanium, the expected floc composition is clay particles and titanium hydroxide flocs. In general, at the end of a run, there was a well-defined froth phase at the top with a clear liquid phase underneath. In some runs, however, some sediment was formed, mainly from Ti flocs. In such cases, sampling the clear phase was conducted carefully to prevent sediment intrusion. The solid removal efficiency was obtained under different operating conditions of (i) applied potential in V/SHE, (ii) residence time in min, and (iii) salt concentration of solutions in mol/L as indicated in Table A3 and Table A4 and Figure A1 and Figure A2 in Appendix A for NaCl and KCl solutions, respectively. The effect of the concentration of NaCl and KCl shown in Figure 5 indicates that for an applied potential of 10 V/SHE and a residence time of 10 min, Ti flocs are generated preferentially in NaCl solutions relative to Ti flocs and KCl, a phenomenon that is explained below:

For NaCl electrolytes, the high *R_MF_* values observed at runs with 20 V/SHE and 20 min indicates large amounts of Ti flocs that were efficiently removed. In contrast, at the lowest *R_MF_* values observed at 10 V/SHE and 10 min, noticeable sediment at the reactor bottom indicates a condition by which a residence time of 10 min is not enough to achieve bubble–particle attachment and removal to the froth layer; in fact, a similar condition, but with a 20 min residence time and a sharp increase in *R_MF_* value, is produced. Interestingly, at this condition, *R_TSS_* values are significantly higher than those of *R_MF_*, which indicates both that the sediment is predominantly Ti flocs and that Ti flocs and clay particles do not attach themselves; in other words, clay particles may be removed in the absence of Ti flocs. However, a synergistic effect between Ti flocs and kaolinite particles to improve removal efficiency is also suggested from the largest *R_TSS_* values observed in combination with the largest *R_MF_* values at 20 V/SHE of applied potential. The effect of residence time on removal is related to the time to achieve the saturation condition or the time to achieve a good production rate of Ti ions and H_2_ bubbles for the coagulation process to work well. Bubbles released from the Ti electrodes at the early reactor operation may be dissolved in solution while the gas concentration at the bulk phase is below saturation. 

The effect of salt concentration had a moderate impact on the removal of kaolinite from the solution. Figure 5A shows that the *R_TSS_* efficiency was slightly higher at 0.1 mol/L NaCl than at 0.01 mol/L NaCl. The highest recovery of 91% kaolinite was achieved at 0.1 mol/L NaCl, a residence time of 20 min, and a potential of 20 V/SHE. The same behavior was observed at 10 V/SHE, with average values of 59.3% and 64.4% for 0.01 and 0.1 mol/L, respectively. For KCl solutions, results of which are shown in Figure 5B, the behavior is somewhat different. In general, *R_MF_* values are significantly lower than those corresponding to NaCl solutions, which indicates a lower floatability for Ti flocs in KCl solutions. The better floatability for Ti flocs in NaCl explains the higher *R_TSS_* values compared to those in KCl in terms of a floc–bubble aggregate formation mechanism that improves particle collision and attachment with subsequent separation from the froth layer. The highest *R_TSS_* values for KCl being observed only at runs with 20 V/SHE, irrespective of the *R_MF_* value, denotes a low floc–particle interaction, and as a consequence, particle–bubble attachment is the main mechanism to explain particle floatability. However high the *R_MF_* values are, as is the case for runs at 20 V/SHE, the floc–particle interaction positively affects the removal of particles. 

The influence of residence time on the removal efficiency of Ti flocs is like that of NaCl, although lower RMF values for KCl are observed. The effect of KCl concentration significantly impacted removing kaolinite from the solution only at a high residence time. The highest recovery of 81% kaolinite was achieved at 0.1 mol/L KCl, a residence time of 20 min, and a potential of 20 V/SHE. In contrast, at 10 V/SHE, average values of 66.9% and 49.4% for 0.01 and 0.1 mol/L, respectively, were observed. 

**Figure A3:**
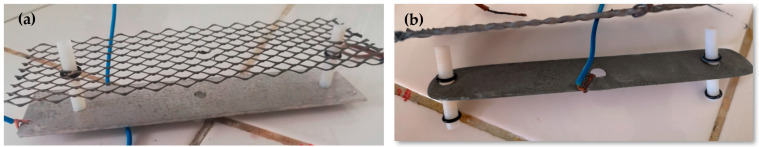
EF electrode. (**a**) Cathode of Ti mesh (over) before EF process. (**b**) Anode of pure Al (under) after EF process.

Kaolinite recoveries over 100% were attributable to the effect of the dissolution of the Ti anode according to the mechanism shown in Equation (8), generating a weight loss of the electrode. The reactive ions from the Ti anode were removed together with the kaolinite. This effect is explained in more detail in the next section. The preceding is complemented with the results previously obtained by the authors when using AISI 316L and Al materials, which demonstrated poor performance under the same potential applied and salinity concentration but were not presented in this work; see the referential photo exhibited in Figure A3.

### 3.4. Result of Electroflotation Process Using Titanium Electrode in 0.1 M NaCl

Ti electrodes play a fundamental role in the removal of kaolinite. The generated bubbles and flocs are created according to electrochemical reactions shown in Equations (3)–(6) [53,54,55,56,57]. The microbubbles of H_2_ and O_2_ generated on the surface of the Ti electrodes in contact with a saline electrolyte during the EF process proceed via specific mechanisms of H_2_ evolution reaction (*HER*) and O_2_ evolution reaction (*OER*) in Equations (3) and (5), respectively. On the other hand, the electrocoagulation (EC) process is related to the dissolution of the anodic electrode according to the metal oxidation reaction shown in Equation (4) [54]. For more details on the *HER* and *OER* mechanisms, refer to Appendix B.

At the cathode (flotation):

(3) $${4H}_{2}O+{4e}^{-}\to {2H}_{2}+4{OH}^{-}$$

**Figure 6:**
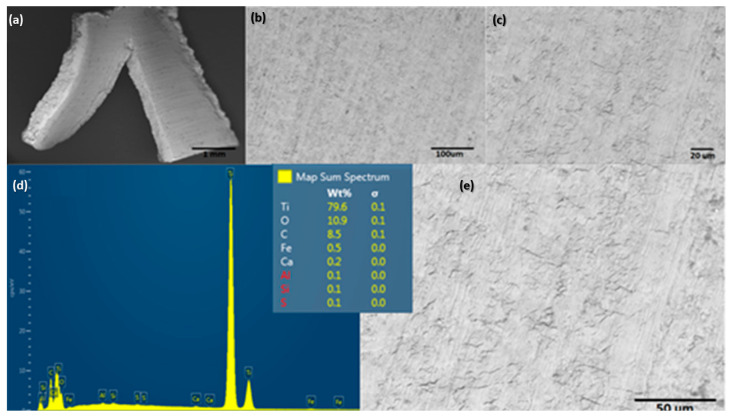
Surface characterization of electrode material used for evaluating the EF/EC process for removal of ultrafine clays: (**a**–**c**,**e**) show a different magnification of SEM, and (**d**) shows EDS spectra.

The material electrodes used for seawater splitting in this experiment are shown in Figure 6. This image shows the superficial condition of the Ti mesh electrodes from Balance World Inc. before contact with the saline electrolyte and applied high potential for EF/EC process. This set of SEM images shows the Ti alloy with a mass composition of 79.6% Ti, 10.9% O, and 8.5% C. Other elements, such as Fe, Ca, Al, Si, and S are considered metal impurities.

**Figure 7:**
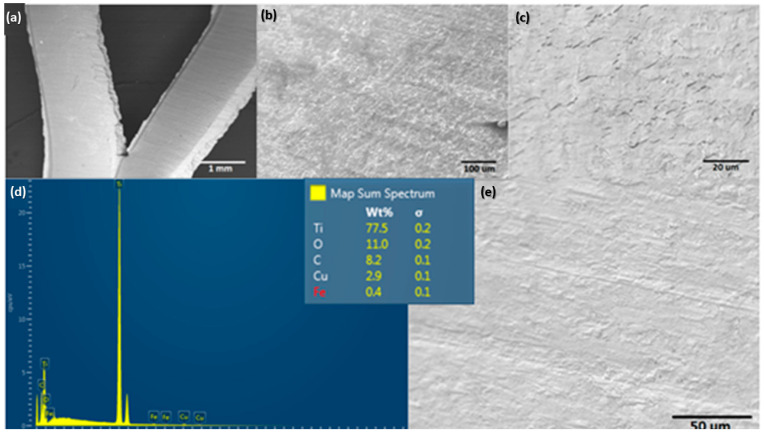
Surface characterization of the cathodic electrode after an EF process at 20 V/SHE. (**a**–**c**,**e**) show different magnifications of SEM, and (**d**) shows EDS spectra.

**Figure 8:**
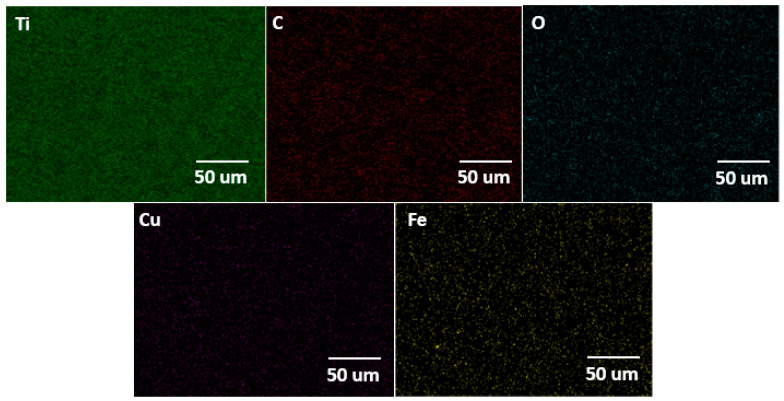
EDS elemental mapping of the cathodic electrode after an EF process at 20 V/SHE.

The surface morphology of the Ti alloy used as a cathodic electrode did not have significant changes concerning the initial surface conditions of the material. However, the EDS elemental mapping indicates that the chemical composition initially changed after the material operated at cathodic potentials (see Figure 7). In the same way, Figure 8 shows the EDS mapping for determining the elemental modification of a Ti alloy as a cathode electrode after EF process for clays recovery in the saline electrolyte. Elements such as Fe, Ca, Al, Si, and S disappeared in the initial conditions and could pass into the electrolyte due to the release of H_2_ microbubbles and the simultaneous pathway reactions during the ORR on the surface cathode electrode.

**Figure 9:**
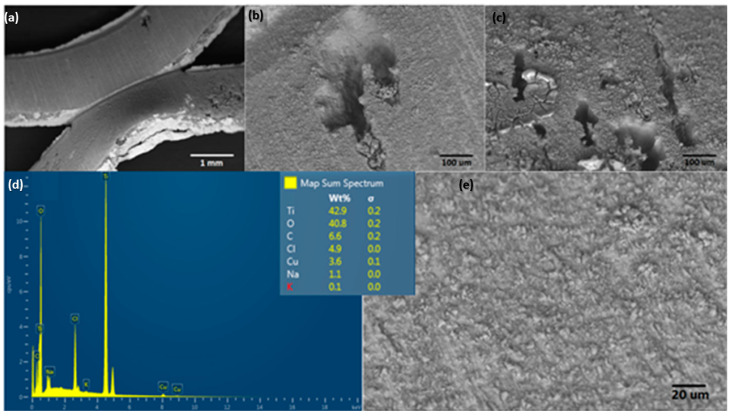
Surface characterization of the anodic electrode after an EF process at 20 V/SHE with a 0.1 M NaCl solution. (**a**–**c**,**e**) show different magnifications of SEM, and (**d**) shows EDS spectra.

An evidential difference showing the effect of salinity on the surface electrode is presented in Figure 9. This image shows pitting corrosion that causes an irregular and empty zone over the electrode. A potential cause could be the anodic potential applied on the Ti electrode generating pitting corrosion (see Equations (4)–(7)), with subsequent reactive ions, such as Ti^2+^, Ti^3+^, and Ti^4+^ that evolve into TiO_2_ as a passive film over the electrode surface. The solid TiO_2_ would finally be formed on the Ti electrode surface using the following mechanism [58]:

At the anode (coagulation):

(4) $$Ti\to {Ti}^{4+}+{4e}^{-}$$

2H_2_O_(l)_ → 4H^+^(aq) + O_2_(g) + 4e^−^ (5)

The mechanism for Ti(OH)_4_ formation:

(6) $${Ti}^{4+}+4{OH}^{-}\to Ti{\left(OH\right)}_{4}$$

The effect of Cl^−^ ions on Ti electrode dissolution [59]:

(7) $${Ti}_{x}O_{y}+{2Cl}^{-}\to {Ti}_{x}O_{y}{Cl}_{2}+{2e}^{-}$$

**Figure 10:**
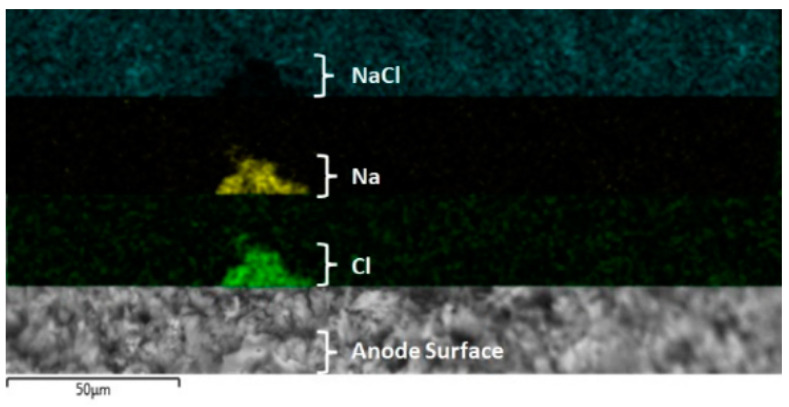
EDX elemental mapping of the anodic electrode after an EF/EC process at 20 V. Up to down: black color = NaCl electrodeposition, yellow color = sodium, green color = chloride, and SEM image of zone analyzed.

The dissolution of titanium (Equation (7)) and pitting corrosion suggested by the authors is summarized as shown in Figure 10, where the NaCl deposition over the titanium electrode is evidenced. In this figure (see up to down), a black shadow pyramid form is shown, which was coincident with the Na (yellow color) and Cl (green color) elements detected by EDS mapping analysis.

Another characteristic evidenced during the development of the experiment was that the number of microbubbles of H_2_ and O_2_ released from the Ti electrodes continuously increased. It is assumed that the effect of the Cl^−^ ion on the anode electrode is similar when using salt solutions, either NaCl or KCl; a decrease in Cl^−^ ion concentration in the electrolyte could be due to the oxidation to Cl_2_ gas or hypochlorite formation over the Ti electrode interface [58]. The global electrochemical reactions generate H_2_ and O_2_ evolution together with the formation of Ti(OH)_4_, which could interact with ultrafine clay particles according to what is described in the literature [53], which improves the removal efficiency of kaolinite particles. The formation of the electrochemical coagulant is the product of reactions on the cathode surface according to the general form such as M^m+^ + m(OH)^−^ → M(OH)_m_, where M = Fe, Al, Mg, Ti, etc. [60], and m is the oxidation state, respectively, which depends on the species present in the electrolyte and the behavior of the cathode and anode materials in contact with saline media that promote the dissolution of the anode [54,61,62,63,64,65,66,67]. The anodic potential applied on the Ti electrode generates pitting corrosion, with subsequent reactive ions, such as Ti^2+^, Ti^3+^, and Ti^4+^ that evolve into TiO_2_ as a passive film on the electrode surface. The solid TiO_2_ would finally be formed on the Ti electrode surface based on the following mechanism [58].

(8) $$Ti\overset{{2e}^{-}}{\to }{Ti}^{2+}\overset{e^{-}}{\to }{Ti}^{3+}\overset{e^{-}}{\to }{Ti}^{4+}$$

The experimental measurements of weight loss of Ti electrodes at 20 V/SHE of applied potential and 20 min residence time were 0.024% and 4.702% for the cathodic and anodic electrodes, respectively. The current density was 23.05 A/m^2^, calculated using Faraday’s law, according to Equation (9):

(9) $$i=\frac{m_{l}\cdot n\cdot F}{A\cdot M\cdot t}$$

where $m_{l}$ is the weight loss of the Ti anode electrode, *n* is the number of electrons transferred in the oxidation reaction, *M* is the atomic weight of the corroded metal g, *A* is the area of the anode electrode in m^2^, and *t* is the electroflotation time. This value is similar to that reported for EC using Al electrodes for phosphate removal [68], but significantly lower when using Fe electrodes for textile wastewater treatment [69]. From the calculated corrosion density value, the estimated molar mass of H_2_ and O_2_ was about 0.19 mmol, which is enough for the reactor volume of 0.25 L to generate bubbles over the saturation concentration in solution at the end of the 20 min residence time.

The results show a promising future for this application using Ti electrodes to remove clay colloids from saline waters in mining processes. The most favorable operating conditions for kaolinite recovery using NaCl and KCl were obtained at 20 V/SHE and 0.1 mol/L, where %*TSS* removal efficiencies were 91.4% and 83.2%, respectively. *XPS* studies are required to confirm the exact contribution of Ti^4+^ as a coagulating agent during the removal of kaolinite clays, in addition to reducing the operating potentials to less than 10 V/SHE.

Although Ti has a higher cost compared to other materials with oxidation potentials greater than Ti (E° = 1.63), as is the case of Al (E° = 1.676) [70], pure Al metal in contact with saline electrolytes shows a noticeably poor resistance to anodic dissolution processes compared to a Ti mesh electrode under the same cell operating conditions. This condition helps to improve the operation of the EF device system since it increases the service life of the anodic electrode, maintaining the efficiency of clay removal over time in the presence of Cl^−^ ions. On the other hand, when Ti operates as an anode in the electrochemical system, it promotes the reduction in the metal dissolution reaction rate in the presence of Cl^−^ ions, enhancing the EF mechanism vs. the EC mechanism [71,72].

## 4. Conclusions

The most favorable operating conditions for kaolinite recovery using NaCl and KCl were obtained at 20 V/SHE and 0.1 mol/L, where %*TSS* removal efficiencies were 91.4% and 83.2%, respectively. The results obtained show a promising future for this application using Ti electrodes for the removal of clay colloids from saline waters in mining processes, understanding of the behavior of this type of electrode materials subjected to direct current without going through a rectifier, in turn, providing the versatility of being able to use renewable energy for its operation and a potential application of these electrode materials on natural seawater. These results provide a novel application of saline water splitting in mineral processing, due to the lack of information about saline EF for the recovery of kaolinite in scientific journals.

## Data Availability

The data that support the findings of this study are available from the corresponding author upon reasonable request.

## Appendix A

**Table A1:** Factors and their levels for experiments used in electroflotation of kaolinite.

Factor	Symbol Factor	Level	
		−1	1
Potential, (V/SHE)	X_1_	10	20
Residence time, (min)	X_2_	10	20
Salinity, (mol/L)	X_3_	0.01	0.1

**Table A2:** Zeta potential experimental data.

	Zeta Potential (±4 mV)		
pH	DIW	NaCl	KCl
3	−13.1	−12.5	18.6
5	−22.31	−19.0	23.2
7	−30.6	−39.5	−31.3
9	−32.5	−34.7	−31.1
10.5	−31.8	−34.3	−30.8
12	−32.9	−25.2	−40.6

**Table A3:** Electroflotation removal of kaolinite on NaCl solutions.

Trial	Time (min)	Voltage	Salinity	Mass Froth (g)	%Rmf	%RTSS
		(V/SHE)	(mol/L)			
1	10	10	0.01	0.029	11.2	59.3
2	10	10	0.1	0.01	3.7	64.4
3	20	10	0.01	0.207	80.9	70.3
4	20	10	0.1	0.226	88.2	76.9
5	10	20	0.01	0.177	69.2	78
6	10	20	0.1	0.226	88.4	87.2
7	20	20	0.01	0.532	207.9	88.9
8	20	20	0.1	0.457	178.7	91.4
					mean	77.05
					s.d.	11.02
					min	59.3
					max	91.4

**Table A4:** Electroflotation removal of kaolinite on KCl solutions.

Trial	Time	Voltage	Salinity	Mass Froth (g)	%Rmf	%RTSS
		(V/SHE)	(mol/L)			
1	10	10	0.01	0.01	4.1	66.9
2	10	10	0.1	0.022	8.6	49.4
3	20	10	0.01	0.107	41.9	71.3
4	20	10	0.1	0.106	41.4	58.1
5	10	20	0.01	0.017	6.7	69.5
6	10	20	0.1	0.007	2.6	83.2
7	20	20	0.01	0.364	142	74.9
8	20	20	0.1	0.173	67.7	80.6
					mean	69.24
					s.d	10.51
					min	49.4
					max	83.2

## Appendix B

The mechanism’s process on the surface electrode.

For alkaline EF-EC conditions, the partial electrochemical reactions for the electrolysis of water are [73,74,75]:

Cathode reaction
  (A1) $$4H_{2}O+4e^{-}\to 2H_{2}+4{OH}^{-}$$
Anode reaction
  (A2) $$4{OH}^{-}\to 2O_{2}+2H_{2}O+4e^{-}$$
Overall electrolysis reactions
  (A3) $${2H}_{2}O+Energy\to {2H}_{2(g)}+O_{2(g)}$$

The mechanism that causes the electroflotation under alkaline conditions for HER and OER could be determined by the following reactions:

The mechanism for H_2_ evolution (HER):
  (A4) $$H_{2}O_{\left(l\right)}+M+e^{-}\to {MH}_{ads}+{OH}^{-}$$
  (A5) $${MH}_{ads}+H_{2}O_{\left(l\right)}+e^{-}↔M+{OH}^{-}+H_{2(g)}$$
  (A6) $$2{MH}_{ads}↔2M+H_{2(g)}$$
The mechanism for O_2_ evolution (OER):
  (A7) $$M+{OH}^{-}\to {MOH}_{ads}+e^{-}$$
  (A8) $${MOH}_{ads}+{OH}^{-}\to {MO}_{ads}+H_{2}O_{\left(l\right)}{+e}^{-}$$
  (A9) $$2{MO}_{ads}\to 2M+O_{2(g)}$$
  (A10) $$MO+{OH}^{-}\to MOOH+e^{-}$$
  (A11) $$MOOH+{OH}^{-}\to M+O_{2(g)}+H_{2}O_{\left(l\right)}+e^{-}$$
  where M is metal and MH, MOH, MO, and MOOH are intermediate products.
The mechanism for Cl_2_ evolution (CER) [76]:
  (A12) $${Cl}_{\left(aq\right)}^{-}+{OH}_{\left(aq\right)}^{-}\to {OCl}_{\left(aq\right)}^{-}+H_{2}O_{\left(l\right)}+2e^{-}$$
  (A13) $$2{Cl}_{\left(aq\right)}^{-}\to {2Cl}_{2}+2e^{-}$$

## References

[1] Brigatti M.F., Galan E., Theng B.K.G. (2006). Chapter 2 Structures and Mineralogy of Clay Minerals. Dev. Clay Sci..

[2] Doi A., Khosravi M., Ejtemaei M., Nguyen T.A.H., Nguyen A.V. (2020). Specificity and affinity of Multivalent Ions Adsorption to Kaolinite Surface. Appl. Clay Sci..

[3] Gräfe M., McFarlane A., Klauber C., Grafe M., Klauber C., McFarlane A.J., Robinson D.J. (2018). Clays and the Minerals Processing Value Chain (MPVC). Clays in the Minerals Processing Value Chain.

[4] Cruz N., Peng Y., Farrokhpay S., Bradshaw D. (2013). Interactions of Clay Minerals in Copper-Gold Flotation: Part 1—Rheological Properties of Clay Mineral Suspensions in the Presence of Flotation Reagents. Miner. Eng..

[5] Molaei N., Hoseinian F.S., Rezai B. (2018). A Study on the Effect of Active Pyrite on Flotation of Porphyry Copper Ores. Physicochem. Probl. Miner. Process..

[6] Shaikh S.M.R., Nasser M.S., Hussein I., Benamor A., Onaizi S.A., Qiblawey H. (2017). Influence of Polyelectrolytes and Other Polymer Complexes on the Flocculation and Rheological Behaviors of Clay Minerals: A Comprehensive Review. Sep. Purif. Technol..

[7] Minh T., Le K., Mäkelä M., Schreithofer N., Dahl O. (2020). A Multivariate Approach for Evaluation and Monitoring of Water Quality in Mining and Minerals Processing Industry. Miner Eng..

[8] Herrera-León S., Lucay F.A., Cisternas L.A., Kraslawski A. (2019). Applying a Multi-Objective Optimization Approach in Designing Water Supply Systems for Mining Industries. The Case of Chile. J. Clean. Prod..

[9] Ikumapayi F.K., Makitalo M., Johansson B., Hanumantharao K. Recycling Process Water in Complex Sulphide Ore Flotationt. Proceedings of the XXVI IInternationa Minerals Processing Congress (IMPC) 2012 Proceednternationa Minerals Processing Congress (IMPC).

[10] Rao K.H., Vilinska A., Chernyshova I.V. (2010). Minerals Bioprocessing: R & D Needs in Mineral Biobene Fi Ciation. Hydrometallurgy.

[11] Mcfarlane A., Yeap K.Y., Bremmell K., Addai-mensah J. (2008). The Influence of Flocculant Adsorption Kinetics on the Dewaterability of Kaolinite and Smectite Clay Mineral Dispersions. Colloids Surf. A Physicochem. Eng. Asp..

[12] Oats W.J., Ozdemir O., Nguyen A.V. (2010). Effect of Mechanical and Chemical Clay Removals by Hydrocyclone and Dispersants on Coal Flotation. Miner. Eng..

[13] Rodrigues R.T., Rubio J. (2007). DAF—Dissolved Air Flotation: Potential Applications in the Mining and Mineral Processing Industry. Int. J. Miner. Process..

[14] Taner H.A., Onen V. (2016). Control of Clay Minerals Effect in Flotation. A Review. E3S Web of Conferences.

[15] Levay G., Smart R.S.C., Skinner W.M. (2001). The Impact of Water Quality on Flotation Performance. J. South. Afr. Inst. Min. Metall..

[16] Romanov A.M. (1998). Electroflotation in Waste Water Treatment: Results and Perspectives. Mineral Processing and the Environment.

[17] Srinivasan V., Subbaiyan M. (1989). Note: Electroflotation Studies on Cu, Ni, Zn, and Cd with Ammonium Dodecyl Dithiocarbamate. Sep. Sci. Technol..

[18] Alexandrova L., Nedialkova T., Nishkov I. (1994). Electroflotation of Metal Ions in Waste Water. Int. J. Miner. Process..

[19] Oussedik S.M., Khelifa A. (2001). Reduction of Copper Ions Concentration in Wastewaters of Galvanoplastic Industry by Electroflotation. Desalination.

[20] Khelifa A., Moulay S., Naceur A.W. (2005). Treatment of Metal Finishing Effluents by the Electroflotation Technique. Desalination.

[21] Merzouk B., Gourich B., Sekki A., Madani K., Chibane M. (2009). Removal Turbidity and Separation of Heavy Metals Using Electrocoagulation-Electroflotation Technique. A Case Study. J. Hazard Mater..

[22] Zouboulis A.I., Matis K.A. (2012). Cadmium Ion Removal by Electroflotation onto Sewage Sludge Biomass. Int. J. Environ. Waste Manag..

[23] Jiménez C., Talavera B., Sáez C., Cañizares P., Rodrigo M.A. (2010). Study of the Production of Hydrogen Bubbles at Low Current Densities for Electroflotation Processes. J. Chem. Technol. Biotechnol..

[24] Fukui Y., Yuu S. (1985). Removal of Colloidal Particles in Electroflotation. AiChE J..

[25] Khosla N.K., Venkatachalam S., Somasundaran P. (1991). Pulsed Electrogeneration of Bubbles for Electroflotation. J. Appl. Electrochem..

[26] Llerena C., Ho J.C.K., Piron D.L. (1996). Effects of PH on Electroflotation of Sphalerite. Chem. Eng. Commun..

[27] Zheng C., Kim D.-S., Park Y.-S. (2017). Turbidity Removal of Kaolin in an Electrocoagulation/Flotation Process Using a Mesh-Type Aluminum Electrode. J. Environ. Sci. Int..

[28] Jiménez C., Sáez C., Cañizares P., Rodrigo M.A. (2016). Optimization of a Combined Electrocoagulation-Electroflotation Reactor. Environ. Sci. Pollut. Res..

[29] Kılıç M.G., Hoşten Ç. (2010). A Comparative Study of Electrocoagulation and Coagulation of Aqueous Suspensions of Kaolinite Powders. J. Hazard Mater..

[30] Cisternas L.A., Gálvez E.D. (2018). The Use of Seawater in Mining. Miner. Process. Extr. Metall. Rev..

[31] Kyzas G.Z., Matis K.A. (2016). Electroflotation Process: A Review. J. Mol. Liq..

[32] Sarkar M.S.K.A., Donne S.W., Evans G.M. (2011). Utilization of Hydrogen in Electroflotation of Silica. Adv. Powder Technol..

[33] Vu T.P., Vogel A., Kern F., Platz S., Menzel U., Gadow R. (2014). Characteristics of an Electrocoagulation-Electroflotation Process in Separating Powdered Activated Carbon from Urban Wastewater Effluent. Sep. Purif. Technol..

[34] Barrera-Díaz C., Bilyeu B., Roa G., Bernal-Martinez L. (2011). Physicochemical Aspects of Electrocoagulation. Sep. Purif. Rev..

[35] Makuei F., Tadesse B., Albijanic B., Browner R. (2018). Electroflotation of Ultrafine Chalcopyrite Particles with Sodium Oleate Collector. Miner. Eng..

[36] Matis K.A., Peleka E.N. (2010). Alternative Flotation Techniques for Wastewater Treatment: Focus on Electroflotation. Sep. Sci. Technol..

[37] Kydros K.A., Gallios G.P., Matis K.A. (1994). Electrolytic Flotation of Pyrite. J. Chem. Technol. Biotechnol..

[38] Bhaskar Raju G., Khangaonkar P.R. (1982). Electro-Flotation of Chalcopyrite Fines. Int. J. Miner. Process..

[39] Hacha R.R., LeonardoTorem M., Gutiérrez Merma A., da Silva Coelho V.F. (2018). Electroflotation of Fine Hematite Particles with Rhodococcus Opacus as a Biocollector in a Modified Partridge–Smith Cell. Miner. Eng..

[40] Liu A., Fan P., Han F., Han H., Li Z., Wang H., Fan M. (2022). Effect of Electroflotation on Quartz and Magnetite and Its Utilization on the Reverse Flotation of Magnetic Separation Concentrate. Miner. Eng..

[41] Tadesse B., Albijanic B., Makuei F., Browner R. (2019). Recovery of Fine and Ultrafine Mineral Particles by Electroflotation—A Review. Miner. Process. Extr. Metall. Rev..

[42] Zhu Z., Zhang W., Cheng C.Y. (2011). A Literature Review of Titanium Solvent Extraction in Chloride Media. Hydrometallurgy.

[43] Bewer G., Debrodt H., Herbst H. (1982). Titanium for Electrochemical Processes. JOM.

[44] Mraz R., Krysa J. (1993). Dimensionally Stables Anodes with a Long Lifetime for Electroflotation. Precision Process Technology.

[45] Ma M., Bruckard W.J., McCall D. (2012). Role of Water Structure-Making/Breaking Ions in the Cationic Flotation of Kaolinite: Implications for Iron Ore Processing. Int. J. Min. Eng. Miner. Process..

[46] Uribe L., Gutierrez L., Laskowski J.S., Castro S. (2017). Role of Calcium and Magnesium Cations in the Interactions between Kaolinite and Chalcopyrite in Seawater. Physicochem. Probl. Miner. Process..

[47] Hu Y., Liu X. (2003). Chemical Composition and Surface Property of Kaolins. Miner. Eng..

[48] Arancibia-Bravo M., Lucay F., Sepulveda F., Cisternas L. (2021). On the Use of Na_2_SO_3_ as a Pyrite Depressant in Saline Systems and the Presence of Kaolinite. Physicochem. Probl. Miner. Process..

[49] Yukselen Y. (2003). Zeta Potential of Kaolinite in the Presence of Alkali. Alkaline Earth and Hydrolyzable Metal Ions. Water Air Soil Pollut..

[50] Zuki N.M., Ismail N., Omar F.M. (2019). Evaluation of Zeta Potential and Particle Size Measurements of Multiple Coagulants in Semiconductor Wastewater. AIP Conference Proceedings.

[51] Dohnalová Z., Svoboda L., Sulcová P. (2008). Characterization of Kaolin Dispersion Using Acoustic and Electroacoustic Spectroscopy. J. Min. Metall. Sect. B Metall..

[52] Gupta V., Miller J.D. (2010). Surface Force Measurements at the Basal Planes of Ordered Kaolinite Particles. J. Colloid Interface Sci..

[53] Tang Y.C., Huang X.H., Wu C.N. (2013). Removal of Arsenic (III) from Drinking Water by Adsorption with Titanium and Ferrous Oxide Nanoparticles. Asian J. Chem..

[54] Ge J., Qu J., Lei P., Liu H. (2004). New Bipolar Electrocoagulation-Electroflotation Process for the Treatment of Laundry Wastewater. Sep. Purif. Technol..

[55] Safwat S.M. (2020). Treatment of Real Printing Wastewater Using Electrocoagulation Process with Titanium and Zinc Electrodes. J. Water Process Eng..

[56] Mansour L.B., Kolsi K., Ksentini I. (2007). Influence of Current Density on Oxygen Transfer in an Electroflotation Cell. J. Appl. Electrochem..

[57] Kotti M., Ksentini I., Ben Mansour L. (2011). Effects of Impurities on Oxygen Transfer Rate in the Electroflotation Process. Desalination Water Treat..

[58] Zhong Y., Yang Q., Li X., Yao F., Xie L., Zhao J., Chen F., Xie T., Zeng G. (2016). Electrochemically Induced Pitting Corrosion of Ti Anode: Application to the Indirect Reduction of Bromate. Chem. Eng. J..

[59] El-Ghenymy A., Alsheyab M., Khodary A., Sirés I., Abdel-Wahab A. (2020). Corrosion Behavior of Pure Titanium Anodes in Saline Medium and Their Performance for Humic Acid Removal by Electrocoagulation. Chemosphere.

[60] Merzouk B., Madani K., Sekki A. (2010). Using Electrocoagulation-Electroflotation Technology to Treat Synthetic Solution and Textile Wastewater. Two Case Studies. Desalination.

[61] Sandbank E., Shelef G., Wachs A.M. (1974). Improved Electroflotation for the Removal of Suspended Solids from Algal Pond Effluents. Water Res..

[62] Morozov A.F., Kon’shina G.I., Morozova V.P. (1981). Electroflotation Extraction of Suspensins Fom Waste Thickeners. Sov. Min..

[63] Manohar C., Kelkar V.K., Yakhmi J.V. (1982). Electroflotation of Colloids without Surfactants. J. Colloid Interface Sci..

[64] Murugananthan M., Raju G.B., Prabhakar S. (2004). Separation of Pollutants from Tannery Effluents by Electro Flotation. Sep. Purif. Technol..

[65] Zuo Q., Chen X., Li W., Chen G. (2008). Combined Electrocoagulation and Electroflotation for Removal of Fluoride from Drinking Water. J. Hazard Mater..

[66] Balla W., Essadki A.H., Gourich B., Dassaa A., Chenik H., Azzi M. (2010). Electrocoagulation/Electroflotation of Reactive. Disperse and Mixture Dyes in an External-Loop Airlift Reactor. J. Hazard Mater..

[67] Tumsri K., Chavalparit O. Optimizing Electrocoagulation-Electroflotation Process for Algae Removal. Proceedings of the 2nd International Conference on Environmenta l Science and Technology IPCBEE.

[68] Attour A., Touati M., Tlili M., Ben Amor M., Lapicque F., Leclerc J.-P. (2014). Influence of Operating Parameters on Phosphate Removal from Water by Electrocoagulation Using Aluminum Electrodes. Sep. Purif. Technol..

[69] Kobya M., Can O.T., Bayramoglu M. (2003). Treatment of Textile Wastewaters by Electrocoagulation Using Iron and Aluminum Electrodes. J. Hazard Mater..

[70] Mechelhoff M., Kelsall G.H., Graham N.J.D. (2013). Electrochemical Behaviour of Aluminium in Electrocoagulation Processes. Chem. Eng. Sci..

[71] Liu T., Tan Y.-J., Lin B.Z.M., Aung N.N. (2006). Novel Corrosion Experiments Using the Wire Beam Electrode, (IV) Studying Localised Anodic Dissolution of Aluminium. Corros. Sci..

[72] Mansouri K., Ibrik K., Bensalah N., Abdel-Wahab A. (2011). Anodic Dissolution of Pure Aluminum during Electrocoagulation Process: Influence of Supporting Electrolyte, Initial PH, and Current Density. Ind. Eng. Chem. Res..

[73] Cao L.-M., Lu D., Zhong D.-C., Lu T.-B. (2020). Prussian Blue Analogues and Their Derived Nanomaterials for Electrocatalytic Water Splitting. Coord. Chem. Rev..

[74] Rajakaruna R.M.P.I., Ariyarathna I.R. (2020). Functionalized Metal-Based Nanoelectrocatalysts for Water Splitting. Handbook of Functionalized Nanomaterials for Industrial Applications.

[75] Mohtashami R., Shang J.Q. (2019). Treatment of Automotive Paint Wastewater in Continuous-Flow Electroflotation Reactor. J. Clean. Prod..

[76] Feng S., Yu Y., Li J., Luo J., Deng P., Jia C., Shen Y. (2022). Recent Progress in Seawater Electrolysis for Hydrogen Evolution by Transition Metal Phosphides. Catal. Commun..

